# Use of prescription analgesic drugs before and after hip or knee replacement in patients with osteoarthritis

**DOI:** 10.1186/s12891-019-2809-4

**Published:** 2019-09-14

**Authors:** Tuomas J. Rajamäki, Pia A. Puolakka, Aki Hietaharju, Teemu Moilanen, Esa Jämsen

**Affiliations:** 1 Faculty of Medicine and Health Technology, Tampere University, FI-33014 Tampere, Finland; 2 Department of Emergency Medicine and Anaesthesia, Tampere University Hospital, PO box 2000, FI-33521 Tampere, Finland; 3 Department of Neurology, Tampere University Hospital, PO box 2000, FI-33521 Tampere, Finland; 4 Coxa, Hospital for Joint Replacement, PO box 652, FI-33101 Tampere, Finland

**Keywords:** Arthroplasty, Analgesics, Drug prescriptions, Opioids, Non-steroidal anti-inflammatory drugs, Acetaminophen

## Abstract

**Background:**

Analgesic drugs are recommended to treat pain caused by osteoarthritis, and joint replacement should decrease the need for them. We aimed to determine the user rates of analgesic drugs before and after joint replacement.

**Methods:**

All patients who underwent a primary hip or knee replacement for osteoarthritis from 2002 to 2013 in a region of 0.5 million people were identified. Patients with revision or other joint replacements during the study period (operation date +/− two years) were excluded, leaving 6238 hip replacements (5657 patients) and 7501 knee replacements (6791 patients) for analyses. Medication data were collected from a nationwide Drug Prescription Register and the prevalence (with its 95% confidence intervals) of acetaminophen, non-steroidal anti-inflammatory drugs (NSAIDs), mild opioids, strong opioids, and medications used for neuropathic pain was calculated in three-month periods two years before and after surgery.

**Results:**

Between two years and three months preoperatively, the proportion of patients who redeemed at least one type of analgesic drug increased from 28% (95% CI, 27–30%) to 48% (47–50%) on hip replacement patients and from 33% (32–34%) to 41% (40–42%) on knee replacement patients. Postoperatively, the proportions decreased to 23% (22–24%) on hip and to 30% (29–31%) on knee patients. Hip replacement patients used more NSAIDs (34% (32–35%) hip vs 26% (25–27%) knee, *p* < 0.001), acetaminophen (14% (13–15%) vs 12% (11–13%), p < 0.001), and mild opioids (14% (13–15%) vs 9% (8–9%), *p* < 0.001) than knee patients preoperatively, but postoperatively hip patients used less NSAIDs (12% (11–13%) vs 16% (15–16%), *p* < 0.001), acetaminophen (9% (8–10%) vs 11% (11–12%), p < 0.001), and mild opioids (5% (5–6%) vs 8% (7–8%), p < 0.001).

**Conclusion:**

Use of analgesic drugs increases prior to joint replacement, and is reduced following surgery. However, a considerable proportion of patients continue to use analgesics in two-year follow-up.

## Background

Pain is the most common symptom of osteoarthritis (OA), and current guidelines for the management of hip and knee OA recommend the use of both non-pharmacological and pharmacological treatment options [1–5]. For those patients who do experience insufficient pain relief with conservative treatment, joint replacement is recommended [6–8]. Nevertheless, 8 to 27% of knee replacement and 5 to 21% of hip replacement recipients suffer from persistent postoperative pain [9].

At present, it is not completely known what proportion of patients use analgesic drugs before and after joint replacement. It has been estimated that nearly half of all knee osteoarthritis patients use pain medication, mostly over-the-counter (OTC) or prescription non-steroidal anti-inflammatory drugs (NSAID) and acetaminophen [10]. Supposedly, the proportion of patients using pain medication **w**ould be greater in those waiting for joint replacement, and it should decrease postoperatively. The proportion of hip and knee replacement recipients using analgesic drugs preoperatively varies from 48 to 94%, depending on methodology of the study [11–15] . The most commonly used drugs are acetaminophen, NSAIDs, and mild opioids [11–15]. These practices are also in accordance with current guidelines for the pharmacological treatment of osteoarthritis [1, 2, 4].

Several recent studies have examined opioid use after joint replacement [16–18], but only a few studies have examined overall analgesic consumption both before and after surgery [11, 13, 15, 19–21]. Prescription data on total hip replacement patients indicate an increase in the use of analgesics (both opioids and non-opioids) during the year before surgery, followed by a peak occurring immediately after surgery and then a decrease during the first postoperative year [13]. In the field of knee replacement, again, previous studies have mainly focused on the risk factors for increased postoperative analgesic consumption [11, 15, 19, 21] or only on the consumption of NSAID’s [20]. Moreover, only a few studies have included medications used for neuropathic pain [11, 13–15, 18], although a remarkable share of joint replacement patients suffer from persistent postoperative pain [9] that may be treated with such drugs. Therefore, research on the consumption of analgesic drugs in joint replacement patients should include all analgesic drugs, and not only acetaminophen, NSAID’s, and opioids.

The aim of this study was to provide a detailed description of consumption trajectories for all prescription analgesic drugs two years before and after hip or knee replacement.

## Methods

### The study population

**Fig. 1:**
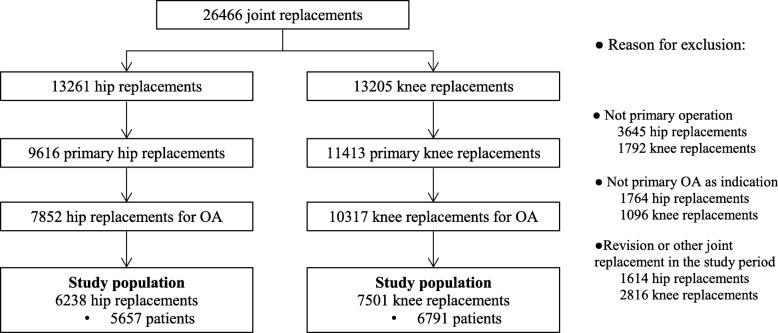
Flowchart

All hip and knee replacement operations in the Pirkanmaa hospital district (population 0.5 million) in Finland are performed in a single orthopedic hospital. Between 2 September 2002 and 31 December 2013, 26,466 operations (13,261 hip replacements and 13,205 knee replacements) were performed on 20,068 patients at the hospital. Preoperative and postoperative information on these patients was collected from the prospective joint replacement database of the hospital. In this study, the inclusion criteria were primary operation and primary osteoarthritis being the indication for surgery. Patients with revisions or other joint replacements during the study period (operation date +/− two years) were excluded, leaving 6238 hip replacements (5657 patients) and 7501 knee replacements (6791 patients) for analyses (Fig. 1).

### Medication data

In Finland, the conservative treatment of osteoarthritis is primarily the responsibility of general practitioners. If conservative treatment is insufficient, patients are referred for consideration for joint replacement [5]. Postoperatively, analgesics are first prescribed by surgeons, whereas general practitioners prescribe analgesics in longer follow-up.

All medications in Finland are dispensed from licensed pharmacies. The Social Insurance Institution of Finland maintains a nationwide Drug Prescription Register that contains information on all prescribed medications that have been dispensed in Finland. In this study, information on the Anatomical Therapeutic Chemical (ATC) code of dispensed drugs, the number of units dispensed (tablets or patches), and the date of purchase was collected from the Drug Prescription Register. Prescription data were linked to joint replacement data using the unique personal identification code assigned to all Finnish residents.

The included medications were classified into subgroups according to their ATC codes as follows: acetaminophen (N02BE01), non-steroidal anti-inflammatory drugs (NSAID) (M01A), mild opioids (N02AA59, N02AX02, N02AE01), strong opioids (N02AA01, N02AA03, N02AA05, N02AB03), medications used for neuropathic pain (N03AX12, N03AX16, N06AA09, N06AA10, N06AX16, N06AX21, i.e., gabapentin, pregabalin, amitriptyline, nortriptyline, venlafaxine, and duloxetine), and any analgesic drug (all previous groups combined). Transdermal buprenorphine patches were classified as mild opioids and transdermal fentanyl patches were classified as strong opioids. Oral buprenorphine and fentanyl as well as oral liquid products of all opioids were excluded because they are used for pain caused by other reasons than osteoarthritis, like cancer pain. Transdermal gel products and oral liquid products of NSAID’s were excluded from this study.

### Statistics

The proportions (with 95% confidence intervals) of patients who redeemed at least one prescription of a medication were calculated in time-periods of three months (90 days) during the 4-year observation period. Three-month periods were chosen because Finnish pharmacies are only allowed to give patients a 90-day supply of prescribed medication at a time. It was recorded, whether a patient had redeemed at least one prescription of a studied medication at a corresponding quartile. The eight quartiles before the operation are referred to as the preoperative period and the eight quartiles after the operation as the postoperative period.

The primary outcome was the detailed description of the analgesic consumption trajectories of the various drug classes two years before and after surgery. An expression ‘user rate’ is used as a synonym for the proportion of patients who redeemed drugs in a specific time-period. Analgesic consumption on late preoperative period (three months preoperatively) was compared to early preoperative consumption (two years preoperatively) and late postoperative (two years postoperatively) consumption. The primary outcome was first analyzed in the whole study population and then in the subgroups according to joint (hip versus knee), gender, and age (< 65 years, 65 to 75 years, > 75 years). Finally, an additional analysis was made to determine the proportions of patients who continued to use analgesic drugs after surgery and the proportion of patients who were new users. In this analysis, the patients were divided into subgroups based on information whether they had redeemed analgesics during the year before surgery or not.

The analyses were performed using IBM SPSS Statistics 24. Bilateral operations were analyzed as one. Parametric variables are presented with mean and standard deviation (SD) and categorical variables with numbers and percentages. Unpaired T-test was used to compare parametric variables and chi-square test was used to compare categorical variables. *P*-values of < 0.05 were considered statistically significant.

## Results

### Study population

The study population was an unselected population-based cohort of OA patients undergoing joint replacement surgery. The mean age was 68.7 years (SD 10.1) and majority (61%) were women (Table 1). Knee replacement patients were older and more often women than hip patients (Table 1). A bilateral operation was performed in 7% of hip and 16% of knee replacements patients.

**Table 1:** Demographic characteristics

	Hip replacement	Knee replacement
Total number, n (%)	6238	7501
Gender, female, n (%)	3319 (53.2%)	5077 (67.7%)
Age, mean (SD), years	67.6 (10.6)	69.7 (9.5)
Bilateral operation, n (%)	418 (6.7%)	1225 (16.3%)
BMI^a^, mean (SD), years	28.2 (4.7)	29.9 (4.8)
Diabetes	473 (7.6%)	744 (9.9%)
Neurodegenerative disease^b^	86 (1.4%)	111 (1.5%)
Cardiac disease^c^	699 (11.2%)	904 (12.1%)
Pulmonary disease	373 (6.0%)	619 (8.3%)
Hypertension	1656 (26.5%)	2491 (33.2%)
Epilepsy	67 (1.1%)	74 (1.0%)
History of malignancy	182 (2.9%)	273 (3.6%)
ASA score		
1	637 (10.2%)	373 (5.0%)
2	2398 (38.4%)	2910 (38.8%)
3	1766 (28.3%)	2590 (34.5%)
4	90 (1.4%)	97 (1.3%)
missing	1347 (21.6%)	1531 (20.4%)

SD = standard deviation

^a^Body mass index; missing information on 866 (13.9%) of hip and 1042 (13.9%) of knee replacement patients

^b^Alzheimer’s or Parkinson’s disease

^c^coronary artery disease, heart failure, chronic arrythmia

### All patients

During the 4-year observation period, 92% (95% CI, 91–93%) of the hip replacement patients and 94% (93–94%) of the knee replacement patients redeemed at least one analgesic drug prescription.

**Fig. 2:**
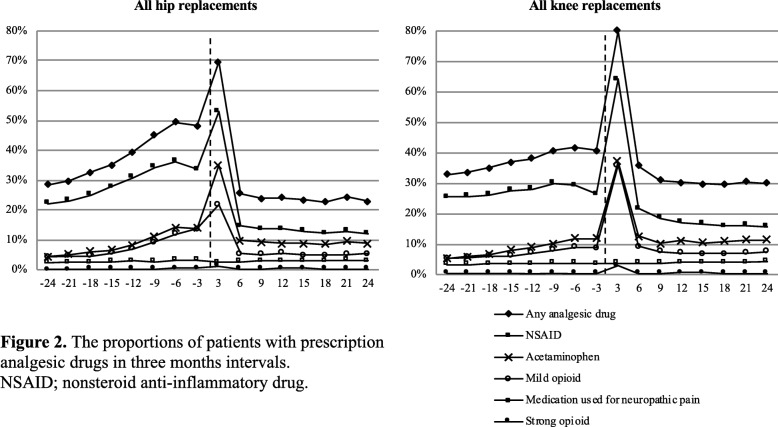
The proportions of patients with prescription analgesic drugs in three months intervals.NSAID;nonstereriod anti-inflammatory drug

The proportion of patients who redeemed at least one type of analgesic drug prescription increased before the surgery (Fig. 2, Table 2). Two years before the surgery, 28% (27–30%) of hip patients and 33% (32–34%) of knee patients redeemed a prescription for at least one type of analgesic drug. By three months before the surgery, the proportions had increased to 48% (47–50%) and 41% (40–42%). After surgery, the use of all analgesics declined and the user rates decreased approximately to the level 2 years before surgery (Fig. 2, Table 2). The preoperative increase was especially attributable to the use of acetaminophen, NSAID’s, and mild opioids in hip patients and to acetaminophen and mild opioids in knee patients. The proportion of patients using NSAIDs decreased in both hip and knee patients in the late preoperative period (Fig. 2).

**Table 2:** Proportions of patients with prescription medication^a^

	Hip replacement	Knee replacement		All patients						
	n (% [95% CI])	n (% [95% CI])	*p*-value	Female n (% [95% CI])	Male n (% [95% CI])	p-value	Age < 65 years n (% [95% CI])	Age 65–75 years n (% [95% CI])	Age > 75 years n (% [95% CI])	*p*-value
N	6238	7501		8396	5343		4817	4807	4115	
Any analgesic drug										
21–24 months preop	1771 (28.4% [27.3–29.5])	2456 (32.7%[31.7–33.8])	< 0.001	2766 (32.9% [31.9–34.0])	1461 (27.3% [26.2–28.5])	< 0.001	1472 (30.6% [29.3–31.9])	1436 (29.9% [28.6–31.2])	1319 (32.1% [30.6–33.5])	0.078
0–3 months preop	3013 (48.3% [47.1–49.5])	3045 (40.6% [39.5–41.7])	< 0.001	3862 (46.0% [44.9–47.1])	2196 (41.1% [39.8–42.4])	< 0.001	2162 (44.9% [43.5–46.3])	2135 (44.4% [43.0–45.8])	1761 (42.8% [41.3–44.3])	0.120
21–24 months postop	1429 (22.9% [21.9–24.0])	2253 (30.0% [29.0–31.1])	< 0.001	2539 (30.2% [29.3–31.2])	1143 (21.4% [20.3–22.5])	< 0.001	1226 (25.5% [24.2–26.7])	1254 (26.1% [24.9–27.3])	1202 (29.2% [27.8–30.6])	< 0.001
Acetaminophen										
21–24 months preop	277 (4.4%[3.9–5.0])	390 (5.2% [4.7–5.7])	0.039	487 (5.8% [5.3–6.3])	180 (3.4% [2.9–3.9])	< 0.001	161 (3.3% [2.8–3.9])	222 (4.6% [4.0–5.2])	284 (6.9% [6.1–7.7])	< 0.001
0–3 months preop	870 (13.9% [13.1–14.8])	894 (11.9% [11.2–12.7])	< 0.001	1203 (14.3% [13.6–15.1])	561 (10.5% [9.7–11.3])	< 0.001	439 (9.1% [8.3–9.9])	628 (13.1% [12.1–14.0])	697 (16.9% [15.8–18.1])	< 0.001
21–24 months postop	546 (8.8% [8.1–9.5])	857 (11.4% [10.7–12.2])	< 0.001	1023 (12.2% [11.5–12.9])	380 (7.1% [6.4–7.8])	< 0.001	313 (6.5% [5.8–7.2])	462 (9.6% [8.8–10.4])	628 (15.3% [14.2–16.4])	< 0.001
NSAID										
21–24 months preop	1387 (22.2% [21.1–23.3])	1916 (25.5% [24.6–26.5])	< 0.001	2125 (25.3% [24.4–26.2])	1178 (22.0% [20.9–23.2])	< 0.001	1224 (25.4% [24.2–26.6])	1139 (23.7% [22.5–24.9])	940 (22.8% [21.6–24.1])	0.014
0–3 months preop	2095 (33.6% [32.4–34.8])	1983 (26.4% [25.4–27.4])	< 0.001	2544 (30.3% [29.3–31.3])	1534 (28.7% [27.5–29.9])	0.047	1664 (34.5% [33.2–35.9])	1455 (30.3% [30.0–31.6])	959 (23.3% [22.0–24.6])	< 0.001
21–24 months postop	749 (12.0% [11.2–12.8])	1168 (15.6% [14.8–16.4])	< 0.001	1276 (15.2% [14.4–16.0])	641 (12.0% [11.1–12.9])	< 0.001	803 (16.7% [15.6–17.7])	675 (14.0% [13.1–15.0])	439 (10.7% [9.7–11.6])	< 0.001
Mild opioid										
21–24 months preop	266 (4.3% [3.8–4.8])	397 (5.3% [4.8–5.8])	0.005	431 (5.1% [4.7–5.6])	232 (4.3% [3.8–4.9])	0.035	233 (4.8%4.2–5.4])	224 (4.7% [4.1–5.3])	206 (5.0% [4.3–5.7])	0.748
0–3 months preop	850 (13.6% [12.8–14.5])	660 (8.8% [8.2–9.4])	< 0.001	963 (11.5% [10.8–12.2])	547 (10.2% [9.4–11.1])	0.024	588 (12.2% [11.3–13.1])	488 (10.2% [9.3–11.0])	434 (10.5% [9.6–11.5])	0.003
21–24 months postop	334 (5.4% [4.8–5.9])	571 (7.6% [7.0–8.2])	< 0.001	631 (7.5% [7.0–8.1])	274 (5.1% [4.5–5.7])	< 0.001	295 (6.1% [5.5–6.8])	305 (6.3% [5.7–7.0])	305 (7.4% [6.6–8.2])	0.035
Strong opioid										
21–24 months preop	7 (0.1% [0.03–0.2])	25 (0.3% [0.2–0.5])	0.007	23 (0.3% [0.2–0.4])	9 (0.2% [0.1–0.3])	0.211	10 (0.2% [0.1–0.3])	10 (0.2% [0.1–0.3])	12 (0.3% [0.1–0.5])	0.647
0–3 months preop	29 (0.5% [0.3–0.6])	29 (0.4% [0.3–0.5])	0.481	35 (0.4% [0.3–0.6])	23 (0.4% [0.3–0.6])	0.905	18 (0.4% [0.2–0.6])	22 (0.5% [0.3–0.7])	18 (0.4% [0.2–0.6])	0.804
21–24 months postop	15 (0.2% [0.1–0.4])	31 (0.4% [0.3–0.6])	0.081	35 (0.4% [0.3–0.6)	11 (0.2% [0.1–0.3])	0.037	12 (0.2% [0.1–0.4])	16 (0.3% [0.2–0.5])	18 (0.4% [0.2–0.6])	0.307
Medication used for neuropathic pain										
21–24 months preop	144 (2.3% [1.9–2.7])	233 (3.1% [2.7–3.5])	0.004	290 (3.5% [3.1–3.8])	87 (1.6% [1.3–2.0])	< 0.001	145 (3.0% [2.5–3.5])	126 (2.6% [2.2–3.1])	106 (2.6% [2.1–3.1])	0.370
0–3 months preop	192 (3.1% [2.7–3.5])	262 (3.5% [3.1–3.9])	0.176	334 (4.0% [3.6–4.4])	120 (2.2% [1.9–2.6])	< 0.001	184 (3.8% [3.3–4.4])	154 (3.2% [2.7–3.7])	116 (2.8% [2.3–3.3])	0.027
21–24 months postop	181 (2.9% [2.5–3.3])	317 (4.2% [3.8–4.7])	< 0.001	370 (4.4% [4.0–4.9)	128 (2.4% [2.0–2.8])	< 0.001	175 (3.6% [3.1–4.2])	165 (3.4% [2.9–4.0])	158 (3.8% [3.3–4.4])	0.591

^a^Table presents number (%) of patients who redeemed prescription medication in two time periods preoperatively (21–24 months, 0–3 months) and one time period postoperatively (21–24 months)

In supplementary analysis, the drug use was analyzed in one year periods, and 73% (72–74%) of hip patients and 67% (66–68%) of knee patients redeemed at least one prescription one year before the surgery (Appendix 1).

A peak in the number of patients who redeemed medication was seen in the immediate postoperative period for all analgesic drugs. After the immediate postoperative period, fewer hip patients redeemed analgesics (except strong opioids) than knee patients: Two years after surgery any analgesics were redeemed by 23% of hip and 30% of knee patients (*p* < 0.001), acetaminophen by 9% of hip and 11% of knee patients (p < 0.001), NSAID by 12% of hip and 16% of knee patients, mild opioid by 5% of hip and 8% of knee patients (p < 0.001) and medication used for neuropathic pain by 3% of hip and 4% of knee patients (p < 0.001) (Fig. 2).

The most common analgesic drugs throughout the study period were NSAIDs, except on patients > 75 years old who redeemed more often acetaminophen than NSAID’s in the late postoperative period (Table 2). Three months before the surgery, 34% (95% CI: 32–35%) of hip replacement patients and 26% (25–27%) of knee patients redeemed NSAIDs, followed by acetaminophen (14% (13–15%) of hip patients and 12% (11–13%) of knee patients), mild opioids (14% (13–15%) and 9% (8–9%)), medications used for neuropathic pain (3% (3–4%) and 4% (3–4%)), and strong opioids (0.5% (0.3–0.6%) and 0.4% (0.3–0.5%), respectively) (Table 2).

Those patients who had redeemed analgesics preoperatively also redeemed them more often postoperatively compared to those patients who had not redeemed analgesics preoperatively (*p* < 0.001) (Table 3). Of the patients who had not redeemed analgesics three months preoperatively, a higher proportion of knee patients (19%) than hip replacement patients (13%) redeemed any analgesics two years postoperatively (p < 0.001).

**Table 3:** The proportions of hip and knee replacement patients who continued to redeem analgesics^a^ or were new to redeem analgesics^b^ postoperatively

	Preoperative	Proportion of patients who redeemed analgesics postoperatively			
	0–3 months preop	0–3 months postop	3–6 months postop	9–12 months postop	21–24 months postop
Any analgesic drug					
Preoperative use^a^	6058	5141 (84.9%)	2933 (48.4%)	2591 (42.8%)	2424 (40.0%)
No preoperative use^b^	7681	5205 (67.8%)	1331 (17.3%)	1187 (15.5%)	1258 (16.4%)
		p < 0.001	p < 0.001	p < 0.001	p < 0.001
Acetaminophen					
Preoperative use^a^	1764	1162 (65.9%)	669 (37.9%)	569 (32.3%)	507 (28.7%)
No preoperative use^b^	11,975	3796 (31.7%)	881 (7.4%)	812 (6.8%)	896 (7.5%)
		p < 0.001	p < 0.001	*p* < 0.001	p < 0.001
NSAID					
Preoperative use^a^	4078	2947 (72.3%)	1440 (35.3%)	1211 (29.7%)	1032 (25.3%)
No preoperative use^b^	9661	5181 (53.6%)	1102 (11.4%)	912 (9.4%)	885 (9.2%)
		p < 0.001	p < 0.001	*p* < 0.001	p < 0.001
Mild opioid					
Preoperative use^a^	1510	903 (59.8%)	476 (31.5%)	414 (27.4%)	384 (25.4%)
No preoperative use^b^	12,229	3159 (25.8%)	549 (4.5%)	452 (3.7%)	521 (4.3%)
		p < 0.001	p < 0.001	p < 0.001	p < 0.001
Strong opioid					
Preoperative use^a^	58	34 (58.6%)	24 (41.4%)	24 (41.4%)	16 (27.6%)
No preoperative use^b^	13,681	251 (1.8%)	30 (0.2%)	39 (0.3%)	30 (0.2%)
		p < 0.001	p < 0.001	p < 0.001	p < 0.001
Medication used for neuropathic pain					
Preoperative use^a^	454	278 (61.2%)	275 (60.6%)	283 (62.3%)	246 (54.2%)
No preoperative use^b^	13,285	165 (1.2%)	168 (1.3%)	197 (1.5%)	252 (1.9%)
		p < 0.001	p < 0.001	p < 0.001	p < 0.001

^a^The number of patients who had redeemed drugs 3 months preoperatively. The following numbers represent how many of them continued to redeem drugs in different time periods postoperatively (i.e. old users)

^b^The number of patients who had not redeemed drugs 3 months preoperatively. The following numbers represent how many of them redeemed drugs in different time periods postoperatively (i.e. new users)

### Gender

In both operation types, the proportions of patients who redeemed at least one type of analgesic drug, acetaminophen, NSAIDs, and mild opioids, were higher in women than men during the whole study period, with the only exception being the immediate postoperative period (Appendix 2, Table 2). During the study period, the proportion of patients who redeemed medications used for neuropathic pain was higher in women than in men, whereas there were no differences in the use of strong opioids, except on late postoperative period (Appendix 2, Table 2).

### Age

The proportion of patients who redeemed acetaminophen was higher in older age groups (compared to the youngest age group) (Table 2). A higher proportion of younger patients redeemed NSAID’s. In older patients, the proportion of patients who had redeemed at least one type of analgesic drug was higher after surgery compared to younger patients (Table 2). The difference according to age was mostly attributable to hip surgery: two years after surgery 20% of patients < 65 years, 22% of patients 65–75 years, and 28% of patients > 75 years redeemed any analgesics (*p* < 0.001), compared to 31, 29, and 30% (*p* = 0.609), respectively.

## Discussion

This large, regionally all-inclusive study of primary hip and knee replacement patients with primary osteoarthritis shows an increase in the proportion of patients using analgesic drugs before surgery, a peak in use during the immediate postoperative period, and a decrease in the late postoperative period. A surprisingly large share of patients does not use any analgesics preoperatively. However, those who use analgesics preoperatively, are more likely to use them also postoperatively, and one-fifth of hip and almost one-third of knee replacement recipients still use analgesics two years after surgery. These results expand the existing knowledge [13] by showing prescription trajectories have similar pattern in hip and knee replacements but use of analgesics is more common in knee than hip replacement patients after surgery. Use of strong opioids and drugs for neuropathic pain was rare and mostly limited to patients who were using these agents already before surgery.

The observed user rates of analgesic drugs were lower than in earlier studies [12–14, 20]. Variations in study designs may be one explaining factor. Some patients may have used only OTC drugs, leading to underestimation of user rates. Although approximately one-fourth of all NSAIDs are bought over-the-counter [22], the share is probably clearly smaller on patients with chronic painful conditions, like osteoarthritis, because in Finland only small packs of acetaminophen, ibuprofen, and ketoprofen are available OTC and they are more expensive than prescribed analgesics. Furthermore, it is unlikely that the use of OTC drugs could explain the differences of user rates between studied groups of patients. Nevertheless, in accordance with earlier studies [13, 20], there is indeed a surprisingly large proportion (27%) of patients who did not redeem any prescription analgesic drugs during the year before surgery, although pain is the crucial indication for joint replacement [1, 4]. One explanation could be that in some patients the functional impairment and deformity of the operated joint rather than pain has been the main indication for surgery. Additionally, only half of the knee osteoarthritis patients have constant pain, instead, the pain in knee osteoarthritis is typically intermittent weight-bearing pain, and quite often it is unpredictable, too [23, 24].

The proportions of patients using acetaminophen, NSAIDs, and opioids in the late postoperative period were surprisingly high both after hip and knee replacement, again in line with earlier studies [13–15, 17, 18]. The user rates of NSAIDs decreased to a lower level postoperatively than that observed two years preoperatively whereas a small increase was found in acetaminophen, mild opioids, and medications used for neuropathic pain. A possible explanation is persistent postoperative pain which is more common after knee replacement and may affect even one fifth of the patients [9]. Use of analgesics was greater in women who are known to report more osteoarthritis pain and acute procedural pain than men [22, 23]. They also have more persistent postoperative pain [19, 25–27]. Although patients with other joint replacements during the follow-up period were excluded, a multi-joint osteoarthritis and other chronic musculoskeletal conditions are a very likely reason for prolonged analgesic use particularly in the oldest age groups. Unlike some other countries, however, addiction is an unlike explanation, because there has not been an opioid epidemic in Finland and the user rates of opioids were relatively small.

The proportion of patients who redeemed acetaminophen, NSAIDs, or mild opioids increased preoperatively more in hip than in knee replacement patients. Postoperatively, the decrease was higher on hip patients, which has been shown in NSAIDs before [20]. No change in user rates was seen after 9 months in hip replacement patients, but the user rates in knee replacement patients decreased until 12 months postoperatively, which may be related to the longer recovery period after knee replacement. Furthermore, user rates for all the studied medications were higher among knee than hip replacement patients for the whole postoperative follow-up period. A higher prevalence of persistent postsurgical pain following knee replacement may at least partly explain this finding [9].

NSAIDs were the most common analgesic drugs which is in line with the results of previous studies and guidelines [1, 2, 4, 13, 15], although the user rates were somewhat lower than in previous studies [12–14, 20, 28, 29]. Although the efficacy and safety of acetaminophen in the treatment of chronic osteoarthritis pain has been questioned recently [30–32], the drug is safe and widely used among osteoarthritis patients [10, 11, 13–15]. In this study, it was the second most commonly drug used, and like other analgesics, its use was clearly reduced after surgery. Interestingly, approximately twice as many patients redeemed acetaminophen in the late postoperative period than in the early preoperative period. It is possible that especially in the oldest age-groups patients have changed from NSAIDs to acetaminophen because of the risks related to chronic use of NSAIDs [33].

User rates of opioids were lower than expected. Earlier, between 24 and 59% of patients have used opioids (mild and strong opioids have usually been analyzed together) 1 to 2 years before joint replacement [11, 14, 16, 17, 29, 34], whereas in this study the proportions were around 15 and 10% in hip and knee patients three months before surgery, respectively. Similar observation was made also when drug use was analyzed for one year period. The reason for this difference is unclear. Although it should be noted that these results originate from one hospital district, it seems that at population level the overall prevalence of dispensed opioids is lower in Finland (6% in year 2016) [35] than in other Nordic countries (8–12% in women and 6–9% in men in year 2016) [36] or in the United States (17% in year 2017) [37]. Also, the total consumption of opioid analgesics measured in DDD has been lower in Finland [38]. Another explanation might be that opioid prescription has been controlled more tightly in Finland than in some other countries, and a special prescription form which is not possible to copy was needed for strong opioids at the time of this study. All in all, the prevalence of any opioid use was higher in our cohort compared to Finnish population, which is in line with earlier findings (in Sweden 9.6% on individuals without OA compared to 23.7% on individuals with OA) [39].

It is noteworthy, that the number of long-term users of opioids did not increase remarkably after joint replacement, and only a minority of patients were new users of opioids 2 years after hip and knee replacement (3 and 5%, respectively). The consumption of opioids was mostly related to the perioperative phase. Use of strong opioids was rare (less than 0.5%), and only a minority were new users of strong opioids after surgery (0.2%).

Unlike the other analgesics, the user rates of medication used for neuropathic pain did not have a clear association with the time of surgery, suggesting that the indication for the use of these drugs is probably other than osteoarthritis. However, it has been estimated that a neuropathic component is present in one-third of patients with painful osteoarthritis [40, 41] and some patients could benefit from medication used for neuropathic pain. In line with earlier studies [11, 15], the user rates increased during the study period, especially in knee replacement patients. Prolonged postoperative pain is a possible explanation for this observation [9, 42].

There are several strengths in this register study, including the large sample size of an unselected population sample undergoing hip or knee replacement for osteoarthritis. Medication data were extracted from a publicly funded nationwide prescription register with practically complete coverage. Only a few studies have analyzed data of all analgesic drug classes from national registers [11, 13, 15]. We believe that the inclusion of all analgesic drug groups is essential to understand the trends of analgesic consumption before and after joint replacement. However, all these previous studies show the user rates only one year pre- and postoperatively. In this study, the user rates were analyzed two years before the surgery to find out at which point the proportion of users starts to increase, and two years postoperatively to find out whether analgesic use stabilize one year after surgery. Patients with revision or other joint replacement during the follow-up period were excluded, so hip and knee pain on other joints should not hamper the results. All operations were performed in a single orthopedic hospital with a standardized perioperative anesthesia and analgesia.

Register-based studies also have limitations. The pharmacological dispensing data do not inform whether the drug was redeemed because of pain in the operated joint or whether the patient has taken the drug or not. A daily diary of analgesic drug use could give more accurate information on the topic. We did not analyze amounts of drugs used, and inclusion of DDDs (defined daily dose) or OMEQs (oral morphine equivalents) could give more information on this topic. However, the perceptions of pain and the amounts of analgesics used are individual, and the value of reporting proportions of users is that it tells whether there is need or no need for analgesics. We were not able to analyze OTC drugs and topical NSAIDs that may have been used in addition to or instead of prescription analgesics and hence the actual use of analgesics may have been even greater than we observed. In the case of irregular use, purchases of analgesics can occur with longer intervals, and therefore the proportion of users may be underestimated in studies based on register data. A multi-joint osteoarthritis is one confounding factor and we were not able to analyze pain on non-target joints. The intensity of pain and the prevalence of persistent pain could not be evaluated in this study. Although the population was unselected and included all the patients of the region, the generalizability of the results may be limited, because all surgeries were performed in the same hospital, and both indications for joint replacement and prescription practices may vary in different regions even though there are national guidelines for both. It should be noted that due to the large sample size, even minor differences may appear statistically significant.

## Conclusions

In conclusion, the use of analgesic drugs increases before joint replacement surgery, and both hip and knee replacement lead to reduction in the use of pain medications, although the change is lesser after knee replacement. The reductions in the user rates were similar for acetaminophen, NSAIDs and opioids whereas use of medications for neuropathic pain seems to increase slightly. A substantial number of patients continue to use analgesics up to two years after surgery.

## Figures and Tables

**Fig. 3:**
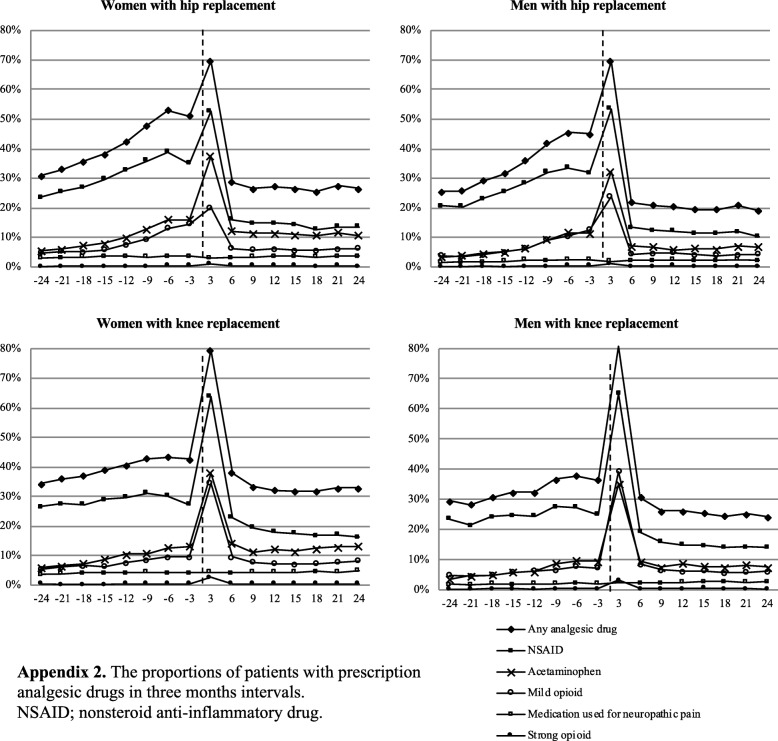
The proportions of patients with prescription analgesic drugs in three months intervals. NSAID; nonsteroid anti-antiflammatory drug

## Data Availability

National legislation and data protection regulations do not allow sharing patient-level materials of this study. Summarized data (like patient numbers) can be provided by corresponding author upon request.

## Appendix 1

**Table 4:** Proportion of patients who redeemed at least one prescription per year

	1. preoperative year		2. preoperative year		1. postoperative year		2. postoperative year		At least one prescription every year	
	N	% (95%CI)	N	% (95%CI)	N	% (95%CI)	N	% (95%CI)	N	% (95%CI)
Hip replacement (*n* = 6238)										
Any analgesic drug	3575	57.3 (56.1–58.5)	4565	73.2 (72.1–74.3)	4784	76.7 (75.6–77.7)	2819	45.2 (44.0–46.4)	1770	28.4 (27.3–29.5)
Acetaminophen	832	13.3 (12.5–14.2)	1643	26.3 (25.3–27.4)	2566	41.1 (40.0–42.4)	1233	19.8 (18.8–20.8)	327	5.2 (4.7–5.8)
NSAID	2996	48.0 (46.8–49.3)	3713	59.5 (58.3–60.7)	3785	60.7 (59.5–61.9)	1814	29.1 (28.0–30.2)	985	15.8 (14.9–16.7)
Mild opioid	662	10.6 (0.99–11.4)	1412	22.6 (21.6–23.7)	1647	26.4 (25.3–27.5)	692	11.1 (10.3–11.9)	155	2.5 (2.1–2.9)
Strong opioid	22	0.4 (0.2–0.5)	43	0.7 (0.5–0.9)	91	1.5 (1.2–1.8)	44	0.7 (0.5–0.9)	4	0.1 (0.00–0.01)
Medication used for neuropathic pain	270	4.3 (3.8–4.8)	338	5.4 (4.9–6.0)	289	4.6 (4.1–5.2)	306	4.9 (4.4–5.4)	105	1.7 (1.4–2.0)
Knee replacement (*n* = 7501)										
Any analgesic drug	4624	61.7 (60.5–62.8)	5052	67.4 (66.3–68.4)	6399	85.3 (84.5–86.1)	4100	54.7 (53.5–55.8)	2678	35.7 (34.6–36.8)
Acetaminophen	1196	15.9 (15.1–16.8)	1795	23.9 (23.0–24.9)	3309	44.1 (43.0–45.2)	1794	23.9 (23.0–24.9)	478	6.4 (5.8–6.9)
NSAID	3863	51.5 (50.4–52.6)	3993	53.2 (52.1–54.4)	5313	70.8 (69.8–71.9)	2653	35.4 (34.3–36.5)	1561	20.8 (19.9–21.7)
Mild opioid	963	12.8 (12.1–13.6)	1323	17.6 (16.8–18.5)	2988	39.8 (38.7–40.9)	1119	14.9 (14.1–15.7)	283	3.8 (3.3–4.2)
Strong opioid	43	0.6 (0.4–0.7)	46	0.6 (0.4–0.8)	246	3.3 (2.9–3.7)	64	0.9 (0.7–1.1)	18	0.2 (0.1–0.4)
Medication used for neuropathic pain	397	5.3 (4.8–5.8)	412	5.5 (5.0–6.0)	504	6.7 (6.2–7.3)	492	6.6 (6.0–7.1)	185	2.5 (2.1–2.8)

NSAID; nonsteroidal anti-inflammatory drug, CI; Confidence interval

## Abbreviations

ATC code: Anatomical Therapeutic Chemical code
CI: Confidence interval
DDD: Defined daily dose
NSAID: Anti-inflammatory drug
OA: Osteoarthritis
OMEQ: Oral morphine equivalent
OTC drug: Over-the-counter drug
SD: Standard deviation
